# Foreword

**DOI:** 10.1017/S0022215116001195

**Published:** 2016-05


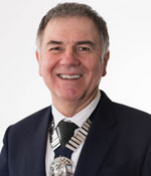
 On behalf of the British Association of Endocrine and Thyroid Surgeons, it is a pleasure to endorse this multidisciplinary document. BAETS represents surgeons who have developed particular expertise in thyroid surgery, regardless of the specialty in which they originally trained. The inclusion of thyroid cancer with upper airway cancers is pragmatic because they share some common features clinically at presentation, particularly the presence of a ‘lump’ in the neck. The most recent British Thyroid Association guidelines for the treatment of thyroid cancer cover the investigation and management of thyroid cancer in depth. BAETS maintains a huge database of outcomes after surgery of the thyroid, both benign and malignant. This satisfies the requirement for surgeons to collect data in line with the requirements of HQIP.

Mark Lansdown BSc, MBBCh, MCh, FRCS

President

British Association of Endocrine and Thyroid Surgeons


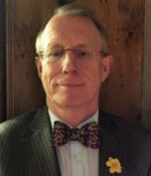
 The United Kingdom is a major player in clinical and basic science research into head and neck cancer, but trying to compare treatment methods is fraught with difficulty, and therefore evidence for one treatment over another is scarce. Due to the complexity and rarity of head and neck cancer, it has always been very difficult to decide what the best treatment is as there are multiple elements to the management.

In 2011, two brave souls decided that the time was right to pull together the great and the good to produce a UK Multidisciplinary Consensus Guideline for Head and Neck Oncology, in an attempt to establish best practice.

This has been the benchmark document for the management head and neck cancer in the UK on which to base our MDT decisions. It was and continues to be truly multidisciplinary.

Over time, treatments are evaluated, so in light of the advances made in radiotherapy delivery and chemotherapeutic options, as well as new technologies e.g. transoral robotic surgery, the time is right to relook at these guidelines and update them,

The British Association of Head and Neck Oncologists represents the multidisciplinary head and neck community within the UK, so as President, I offer once again the grateful thanks of our association to both the editors and the many contributing authors for their tireless efforts in compiling and publishing this essential set of clinical guidelines.

Michael Fardy FFDRCSI, FDSRCS, FRCS

President

British Association of Head and Neck Oncologists


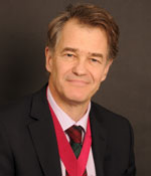
 Guidelines are an essential part of the process of ensuring appropriate treatment is available and provided for patients unfortunate enough to be given a diagnosis of a head and neck malignancy. There is a need to make sure that these guidelines are regularly updated so that our interventions remain up to date and effective, and I am pleased that this has already taken place. As a maxillofacial head and neck surgeon I have seen many changes and improvements, but teamwork, respect and co-operation with colleagues to smooth the patient journey are paramount and have greatly improved. The Liverpool group was lucky enough to have the opportunity to host the European Congress on Head and Neck Oncology in 2014, and demonstrate the high level of team-working in the clinical and research arena. The average head and neck cancer patient has a rocky path to tread, and there is no doubt that such a publication available to all will help them along the way.

Professor James S Brown MD FRCS FDSRCS

President

British Association of Oral and Maxillofacial Surgeons


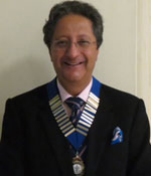
 It is a privilege to write a foreword for this superb document. Once again our head and neck colleagues have demonstrated great collegiality and teamwork to produce an outstanding consensus document. It is also remarkably user friendly and I am sure will provide a superb clinical resource for the benefit of our patients. This approach along with improved data collection and analysis will help keep British surgery at the forefront of care for many years ahead.

Professor Antony A Narula MA MB BChir FRCS FRCS (Ed)

President

British Association of Otorhinolaryngology-Head & Neck Surgery


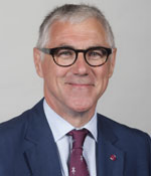
 It is fascinating to compare these excellent, updated guidelines with the old version. Doing so unfolds a story of true multidisciplinary care leading to improved patient outcomes, where each individual in a team knows that they cannot function without the others, and that everyone has skills and strengths to add to the whole for the benefit of the patient under the team's care. As a result of that multidisciplinary care, the treatment of head and neck cancer has changed very much for the better in the last two decades, and I commend those involved in treating these patients for their dedication. In particular, the head and neck surgeons deserve praise for being first on board the National Flap Register, which is a testament to their desire to continuously improve care and outcomes for this complex and heterogenous patient population. Congratulations to all!

Mr Nigel Mercer FRCS

President, 2015–2016

British Association of Plastic, Reconstructive and Aesthetic Surgeons


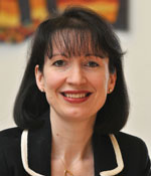
 The Royal College of Pathologists is delighted to endorse this publication and I would like to take the opportunity to thank the authors and editors for all their hard work, particularly Professor Helliwell and Dr Giles, who contributed the pathology content. Members of all the contributing specialties are to be congratulated on the degree of collaboration and consensus reached and the high quality of the resulting document. The latest version of these comprehensive guidelines will support multidisciplinary teams working in head and neck cancer and help them provide the best possible outcome for patients. Additions since the last edition include recent advances in molecular pathology, particularly the development of molecular evaluation for viral-induced cancers. Such quality-assured pathology guidance provides reassurance to clinical teams that pathology information is based on good evidence and has the confidence of pathologists across the UK. Congratulations on an excellent document, which I'm sure will be welcomed by members of all specialties working in this area.

Suzy Lishman FRCPath

President

The Royal College of Pathologists


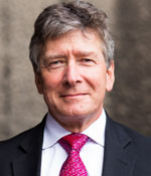
 On behalf of The Royal College of Radiologists I very much welcome the updating of these important multidisciplinary guidelines for head and neck cancer. They provide a valuable resource for all those across many specialties who are involved in the treatment of patients with head and neck cancer and they should continue to be essential reading. The guidelines cover all aspects of head and neck cancer management, from epidemiology and diagnosis through to treatment and outcomes, and I commend the editors and authors – a number of whom are Fellows of the RCR – for this tremendous body of work. I hope this new edition will continue to encourage and support multidisciplinary working and thereby help to improve patient care and ensure the highest possible standards are achieved and maintained.

Dr Giles Maskell

President

The Royal College of Radiologists

